# Anatomic Imaging of the Prostate

**DOI:** 10.1155/2014/728539

**Published:** 2014-08-27

**Authors:** Anil Bhavsar, Sadhna Verma

**Affiliations:** University of Cincinnati Medical Center, Cincinnati, OH 45267-0762, USA

## Abstract

The important role of magnetic resonance imaging (MRI) in the anatomic evaluation, detection, and staging of prostate cancer is well established. This paper focuses on the pertinent embryologic, anatomic, and imaging facts regarding both the normal prostate and the several examples of prostate cancers as well as staging implications. The discussion primarily includes findings related to T2-weighted imaging as opposed to the other functional sequences, including diffusion weighted imaging (DWI) or dynamic contrast enhanced MRI and MR spectroscopic imaging, respectively.

## 1. Introduction

The incidence and mortality of prostate cancer vary substantially worldwide; however, it is the most common noncutaneous malignancy in the western world, affecting approximately 1 out of every 6 men [1]. Although it is the second leading cause of cancer-related death in men (after lung cancer) in the United States, cancer-specific survival is excellent for most patients. In fact, the death rates from prostate cancer have been in significant decline since the mid-1990s. This is likely as a result of earlier diagnosis and treatment. Currently, over 90 percent of patients present with local or locoregional disease due to the widespread use of prostate cancer screening (i.e., use of prostate specific antigen [PSA] or digital rectal examination [DRE]). For most men suspected of having prostate cancer, tissue is obtained through transrectal ultrasound-guided (TRUS) biopsy during which 12 biopsy cores are randomly taken (where the target is generally invisible). However, the role of magnetic resonance imaging (MRI) in the localization and staging of prostate cancer has evolved in the past decade. Advances in MRI show promise for improved detection and characterization of prostate cancer, using a multiparametric approach, which combines anatomical and functional data. For the purposes of this paper, we will discuss prostate MRI techniques, discuss the development/embryology of the prostate gland, and discuss the normal appearance of the prostate gland on T2-weighted imaging (T2WI). Afterwards, a brief discussion regarding prostate cancer and the utility of MRI in staging will ensue.

## 2. MRI Sequences in Prostate Gland Imaging

Multiparametric MRI evaluation of the prostate includes three general components: high-resolution T2WI and at least two functional MRI techniques including diffusion weighted imaging and either MR spectroscopic imaging (MRSI) or dynamic contrast enhanced MRI (DCE-MRI). T2WI provides the best depiction of the prostate's zonal anatomy and capsule. T2WI is used for prostate cancer detection, localization, and staging; however, it alone is not recommended because additional functional techniques improve both sensitivity and specificity [2]. The T2WI sequence should be obtained in 2-3 planes. The axial T2WI sequence should be orthogonal to the rectum and include the entire prostate and seminal vesicles. The phase encoding direction should be oriented left-to-right so that motion artifact (i.e., from bowel) does not overlap the prostate. If desired, an antiperistaltic agent can be given to reduce bowel motion artifacts. Although an endorectal coil is not an absolute requirement, a pelvic phased array coil with a minimum of 16 channels is required [2]. In addition, it is considered good practice to utilize an endorectal coil whenever possible so that the best possible images may be obtained during the examination.

Hemorrhage from prior biopsies can cause artifacts that mimic cancer and thus limit lesion localization and staging. To counter this phenomenon, the time interval between the biopsy procedure and MRI should be at least 4–6 weeks [3]. Also, an initial T1-weighted imaging sequence can be obtained to evaluate biopsy-related hemorrhage. Significant hemorrhage should preclude the remainder of the study, and the individual can be rescheduled 4–6 weeks later to allow for resolution of the hemorrhage [2].

As previously mentioned, T1-weighted imaging sequences should be obtained to evaluate prior biopsy-related hemorrhages. However, additional utilities for this sequence include evaluating regional lymphadenopathy and osseous metastases within the pelvis.

## 3. Prostate Embryology and Development of the Prostate Gland

During the third month of gestation, the prostate gland develops from epithelial invaginations from the posterior urogenital sinus. In order for this process to occur normally, the presence of 5α-dihydrotestosterone is required [4]. This molecule is synthesized from fetal testosterone by the action of 5α-reductase and is localized in the urogenital sinus and external genitalia of humans [5]. Deficiencies of 5α-reductase will cause a rudimentary or undetectable prostate in addition to severe abnormalities of the external genitalia, although the epididymides, vasa deferentia, and seminal vesicles remain normal [6]. During the prepubertal period, the constitution of the human prostate remains relatively identical; however, it undergoes morphologic changes into the adult phenotype with the beginning of puberty. Ultimately, the gland enlarges to reach the average adult weight of approximately 20 g by 25–30 years of age [4].

## 4. Anatomy of the Prostate Gland

The prostate gland is the largest accessory gland of the male reproductive system. It secretes a thin, slightly alkaline fluid that forms a portion of the seminal fluid. It is composed of glandular and stromal elements which are tightly fused within a pseudocapsule. The inner layer of the prostate capsule is composed of smooth muscle with an outer layer covering of collagen [7]. Nerve supply to the prostate is derived from the prostatic plexus and arterial supply by the branches of the internal iliac artery. Lymphatic drainage from the prostate gland occurs predominantly via the internal iliac nodes.

The prostate gland is located posterior to the lower portion of the symphysis pubis, anterior to the rectum, and inferior to the urinary bladder in the subperitoneal compartment between the pelvic diaphragm and the peritoneal cavity. Classically described as “walnut-shaped,” the prostate gland is conical in shape and surrounds the proximal urethra as it exits from the bladder.

**Figure 1:**
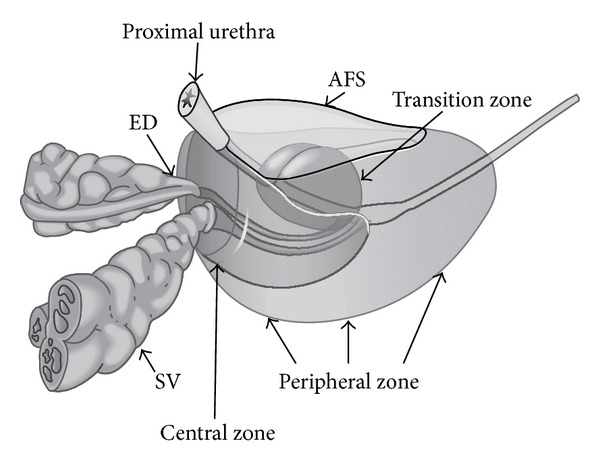
Zonal anatomy of the prostate gland. ED: ejaculatory ducts; SV: seminal vesicles; AFS: anterior fibromuscular stroma.

**Figure 2:**
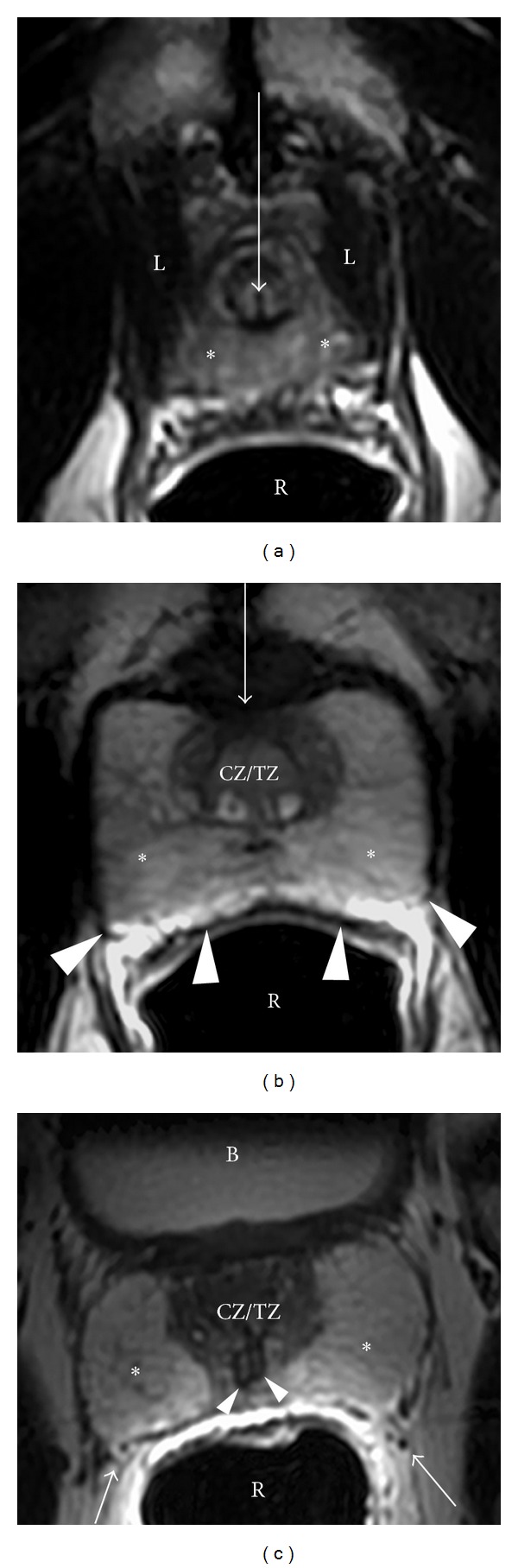
Normal prostate from apex to base in a 54-year-old male (axial plane). (a) Axial T2WI at the level of the apex. The apex consists of the distal part of the prostatic urethra (white arrow) surrounded by high signal intensity, loosely packed peripheral zone tissue (^∗^). The rectum is located posteriorly (R) and is distended by an endorectal coil. The levator ani muscles are located laterally (L). Normal prostate from apex to base in a 54-year-old male (axial plane). (b) At the midgland level, the densely packed central/transition zones are surrounded by high signal-intensity peripheral zone tissue (^∗^), subdivided by several stromal septa which are designated by thin dark T2 signal linear bands. The anterior fibromuscular stroma is a dark T2 band of tissue located anteriorly (arrow). The rectoprostatic angle is depicted posteriorly (arrowheads). Normal prostate from apex to base in a 54-year-old male (axial plane). (c) Base level. The prostatic base is generally composed of almost entirely central zone/transition zone (CZ/TZ); however, a large amount of peripheral zone (^∗^) is noted in this individual. Neurovascular bundles are located posterolaterally (arrows). In addition, the ejaculatory ducts are noted at this level (arrowheads). B: bladder; R: rectum (with endorectal coil).

**Figure 3:**
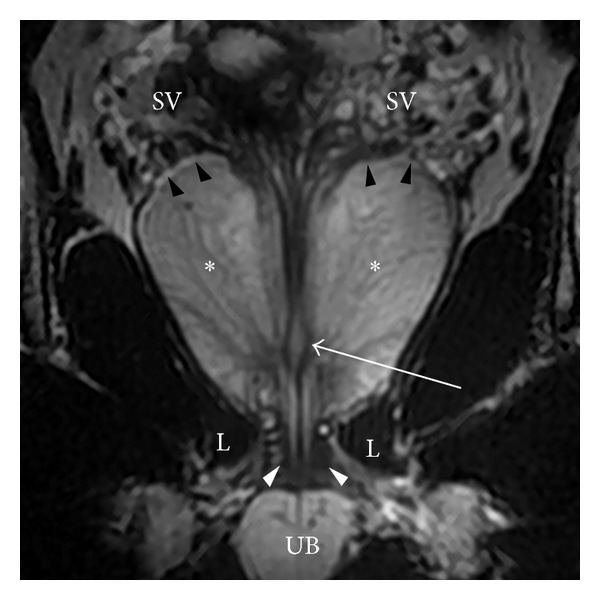
Coronal image through the prostate. At the apex, the distal urethral becomes surrounded by the low signal-intensity external urethral sphincter (white arrowheads) that extends downward to the urethral bulb (UB) and is embraced by the inferomedial aspect of the levator ani muscle (L). In addition, at the midgland, the ejaculatory ducts join the prostatic urethra at the verumontanum, designated by a high signal-intensity structure (arrow). The prostatic-seminal vesicle angle is also seen best in the coronal plane (black arrowheads).

The prostate is divided into four regions, the central zone (CZ), transition zone (TZ), peripheral zone (PZ), and anterior fibromuscular stroma (Figure 1), and is composed of an apex, a base, and anterior, posterior, and inferior-lateral surfaces. Ultimately, the apex is the lower 1/3rd of the prostate gland, the midprostate is the middle 1/3rd of the prostate gland which includes the verumontanum in the midprostatic urethra, and the base is the upper 1/3rd of the prostate just below the urinary bladder (Figures 2 and 3).

The peripheral zone is the larger of the zones, comprising approximately 70% of the glandular tissue. It extends from the base to the apex along the posterior surface and surrounds the distal urethra. In this zone, carcinoma, chronic prostatitis, and postinflammatory atrophy are relatively more common than in the other zones. The peripheral zone contains numerous ductal and acinar elements with sparsely interwoven smooth muscle; thus it is normally of high signal intensity on T2-weighted MRI sequences (Figures 2 and 3).

The central zone is located at the base of the prostate between the peripheral and transition zones and accounts for approximately 25% of the glandular tissue. It is a cone-shaped structure which surrounds the ejaculatory ducts and narrows to an apex at the verumontanum. The verumontanum is a longitudinal mucosal fold that forms an elliptical segment of the prostatic urethra, marking the point where the ejaculatory ducts enter the urethra (Figure 3).

**Figure 4:**
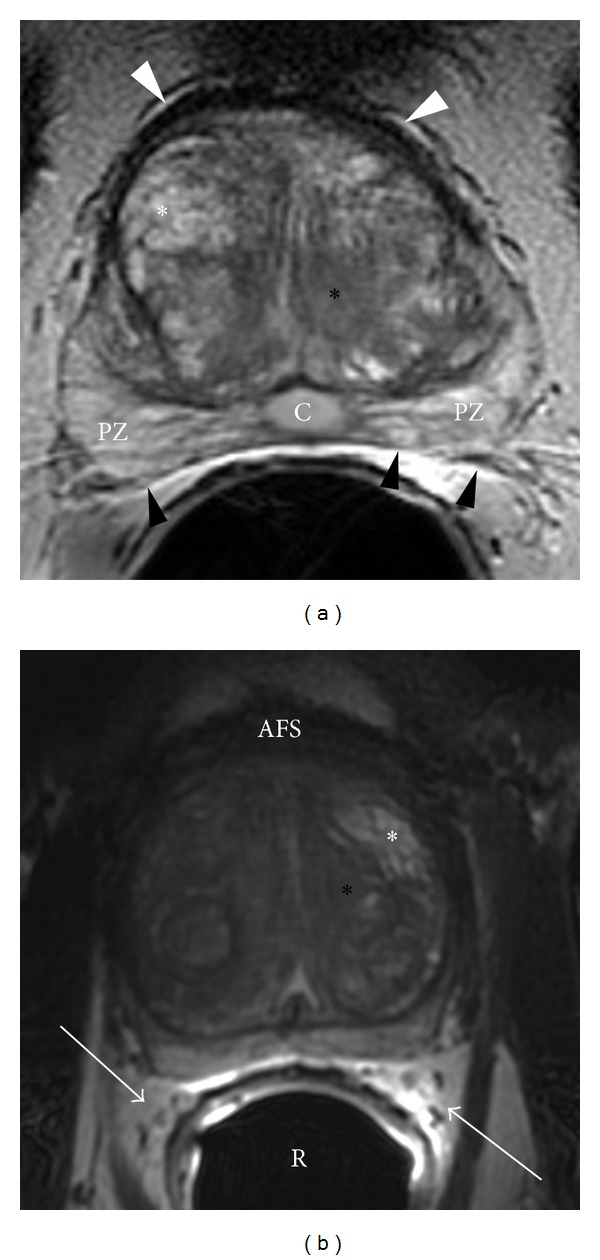
Benign prostatic hyperplasia. (a) Axial T2WI of the prostate at the midgland level. Hyperplasia comprises of both glandular (white asterisk) and stromal (black asterisk) elements. White arrowheads: anterior fibromuscular stroma; black arrowheads: rectoprostatic angle; PZ: peripheral zone; C: incidental Müllerian duct cyst. Benign prostatic hyperplasia. (b) Axial T2WI of the prostate at the midgland level. Hyperplasia comprises of both glandular (white asterisk) and stromal (black asterisk) elements. AFS: anterior fibromuscular stroma; white arrows: neurovascular bundles which are surrounded by fat; R: rectum with endorectal coil.

The transition zone forms only 5% of the glandular tissue and consists of two small lobules of glandular tissue that surround the proximal prostatic urethra just superior to the verumontanum. This is the portion of the glandular tissue that enlarges due to benign prostatic hyperplasia. This hyperplasia does not involve the peripheral zone when it occurs (Figure 4). On MRI, the transition zone usually consists of nodular areas of varying signal intensity, depending on the relative amount of glandular and stromal hyperplasia [8, 9]. Glandular hyperplasia contains relatively more ductal and acinar elements and secretions, resulting in higher signal intensity on T2-weighted MRI sequences (Figure 4). Stromal hyperplasia contains more muscular and fibrous elements, resulting in lower signal intensity (Figure 4). According to the origin of hyperplasia, the term “median lobe hyperplasia” may be used to denote hyperplasia of the periurethral glands [10]. The subsequent compression of the transition zone (also called “surgical pseudocapsule”) may be imperceptible or visible as a faint dark rim, separating the transition gland from the peripheral zone.

The anterior fibromuscular stroma forms the convexity of the anterior external surface and is devoid of glandular tissue and is instead composed of fibrous and smooth muscular elements. Thus, this area is relatively low in signal intensity on T2WI (Figures 2, 3, and 4). The apical half of this area is rich in striated muscle which blends into the gland and the muscle of the pelvic diaphragm. As it extends laterally and posteriorly, it thins to form the fibrous capsule that surrounds the prostate gland. Although the term “capsule” is embedded in the current literature, there is no consensus about the presence of a true capsule [11]. It is usually visible as a sharply demarcated rim at the posterolateral aspects of the prostate on T2WI. In addition, the anterior fibromuscular stroma is separated from the pubic symphysis by Santorini's venous plexus (draining the dorsal veins of the penis) and some ligamentous/fibroadipose tissue in the space of Retzius [12].

These zones have different embryologic origins and can be distinguished by their appearance, anatomic landmarks, biological functions, and susceptibility to pathology (Table 1). Approximately 70% of all prostate cancers arise from the PZ, which is primarily derived from the urogenital sinus. By contrast, a very low incidence of prostate cancer is found in the CZ which is derived from the Wolffian duct. The TZ shares a similar embryologic origin as the PZ; however, the percentage of prostate cancer arising from the TZ is lower, on the order of 25%. This may be explained by the differences in the stromal component of these two zones. The stroma of the TZ is more fibromuscular, and it has been postulated that benign prostatic hyperplasia (BPH), which predominantly arises in the TZ, is a disease of the fibromuscular stroma. This information, including the composition of the various zones, is summarized in Table 1.

As previously mentioned, prostate gland is composed of an apex, a base, and anterior, posterior, and inferior-lateral surfaces. The apex rests on the superior surface of the urogenital diaphragm and contacts the medial surface of the levator ani muscles. At the level of the apex, the prostate gland consists of high T2 signal-intensity peripheral zone tissue (wrapping around the distal prostatic urethra). The ratio of peripheral zone to transition/central zone tissue then gradually decreases upwards to the level of the prostatic base, at which level the prostate gland is almost entirely consisted of mixed signal-intensity central/transition zone tissue. The base is attached to the neck of the bladder and the prostatic urethra enters the middle of it near the anterior surface, which is narrow and convex. The posterior surface is triangular and flat and rests on the anterior wall of the rectum (thus allowing digital palpation for examination). Denonvillier's fascia, a thin, filmy layer of connective tissue, separates the prostate and seminal vesicles from the rectum posteriorly. The inferior-lateral surface joins the anterior surface and rests on the levator ani fascia above the urogenital diaphragm.

Loose connective and adipose tissue containing the periprostatic venous plexus intermixed with arteries, nerves, and lymphatics are located at the posterolateral aspects of the prostate. As a result, these structures are denoted as neurovascular bundles, containing nerve fibers that are important to normal erectile function (Figures 2(c) and 4(b)). Overall, the prostate is an extraordinarily well-innervated organ. The prostate receives both parasympathetic (via the hypogastric and pelvic nerves) and sympathetic innervation (via the peripheral hypogastric ganglion) [13]. Ultimately, these nerves are crucial in regulating the physiology, morphology, and growth maturation of the gland [14–17].

## 5. Seminal Vesicles and Ejaculatory Ducts

**Figure 5:**
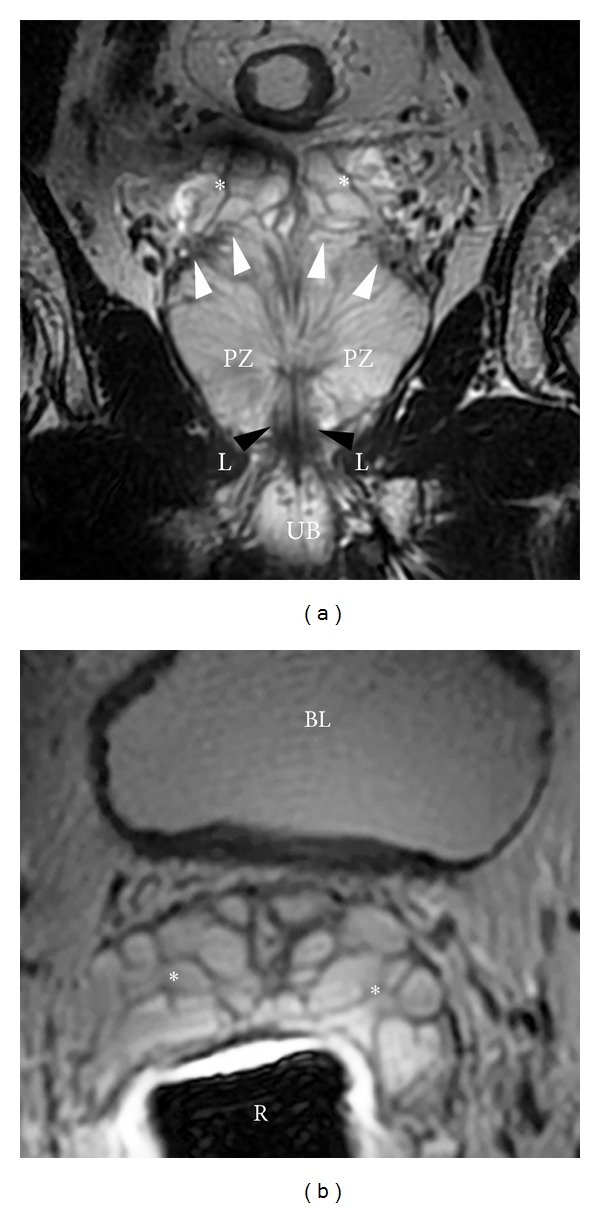
Seminal vesicles. (a) Coronal T2WI showing the prostate and seminal vesicles. Seminal vesicles are high signal-intensity fluid-filled pouches with a low signal-intensity wall, arranged in a grapelike pattern. White arrowheads: prostate-seminal vesicle angle; PB: penile bulb; L: levator ani; black arrowheads: external urethral sphincter; PZ: peripheral zone. Seminal vesicles. (b) Axial T2WI showing the seminal vesicles. Seminal vesicles are high signal-intensity fluid-filled pouches with a low signal-intensity wall, arranged in a grapelike pattern. BL: bladder; R: rectum with endorectal coil.

The seminal vesicles are paired grapelike pouches filled with high signal-intensity fluid on T2WI (Figures 3 and 5). They lay between the bladder and rectum, just caudolateral to the corresponding deferent duct. Their size may vary depending on age and postejaculatory condition [18]. The caudal tip of each seminal vesicle joins the corresponding deferent duct to form the ejaculatory duct, which is enveloped in a thick low T2 signal-intensity muscular coat and traverses the central zone of the prostate to terminate at the verumontanum (Figures 2(c), 3, and 5).

## 6. Prostate Cancer

**Figure 6:**
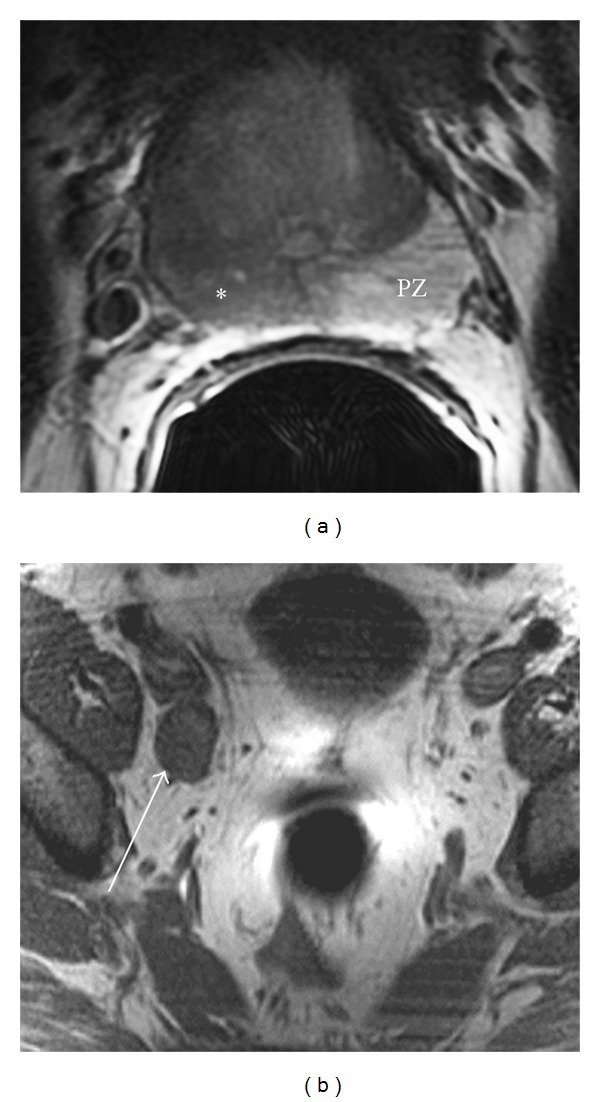
50-year-old male with prostate cancer. (a) Axial T2WI showing low signal within the rightward aspect of the peripheral zone (^∗^). Note the normal-appearing contralateral peripheral zone (PZ) comprised of glandular elements. Also, the right rectoprostatic angle appears slightly ill-defined, although no definite bulge is seen. 50-year-old male with prostate cancer. (b) Axial T1WI showing an enlarged right external iliac chain lymph node, a regional lymph node according to the TNM staging system. This would comprise N1 disease, which would fall under Stage IV category.

**Figure 7:**
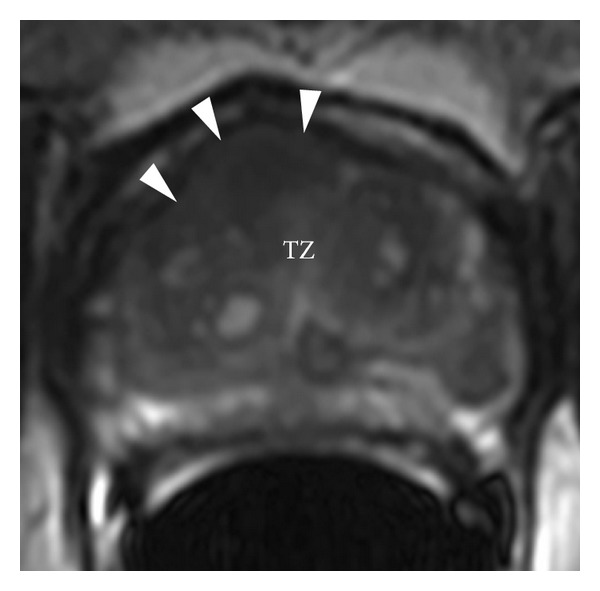
Prostate cancer. Axial T2WI showing a right anterior transitional zone tumor within the midgland, likely also involving a portion of the anterior fibromuscular stroma. Notice that the tumor creates a slight anterior bulge (arrowheads). According to the TNM staging system, this tumor involves less than 50% of a single lobe of the prostate, thus indicating a T2 tumor.

Prostate cancer typically presents as a round or ill-defined low signal-intensity focus in the peripheral zone on T2WI. Since the majority of all prostate carcinomas arise in the peripheral zone, many of them can be readily detected within the high signal-intensity background of the loosely packed normal peripheral zone glandular tissue (Figure 6). Unfortunately, this sign is by no means specific. Other entities such as chronic prostatitis, hemorrhage, scar tissue, atrophy, prostate intraepithelial neoplasia, and posttreatment changes can mimic cancer on T2WI. Tumors located in the TZ are even more challenging to detect given that the low T2 signal intensity densely packed stromal elements and BPH nodules in the TZ overlap with prostate cancers (Figure 7) [19]. TZ tumors are often shown as a homogenous signal mass with indistinct margins (“erased charcoal sign”).

**Figure 8:**
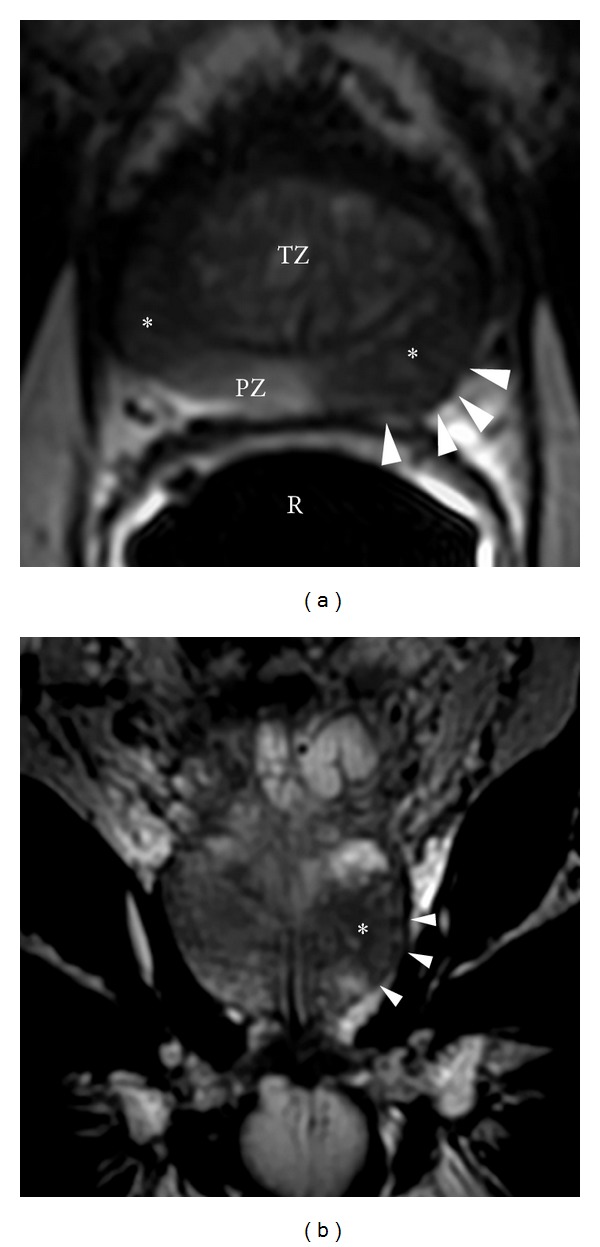
Prostate cancer. (a) Axial T2WI showing multifocal peripheral zone tumors (^∗^). Notice the subtle asymmetric posterolateral bulging along the left rectoprostatic angle which would be concerning for possible extracapsular extension (arrowheads). TZ: transition zone with BPH changes; R: rectum with endorectal coil; PZ: normal-appearing peripheral zone. Prostate cancer. (b) Coronal T2WI through the central portion of the left posterolateral peripheral zone tumor (^∗^). Again notice the subtle asymmetric posterolateral bulging which would be concerning for possible extracapsular extension (arrowheads).

**Figure 9:**
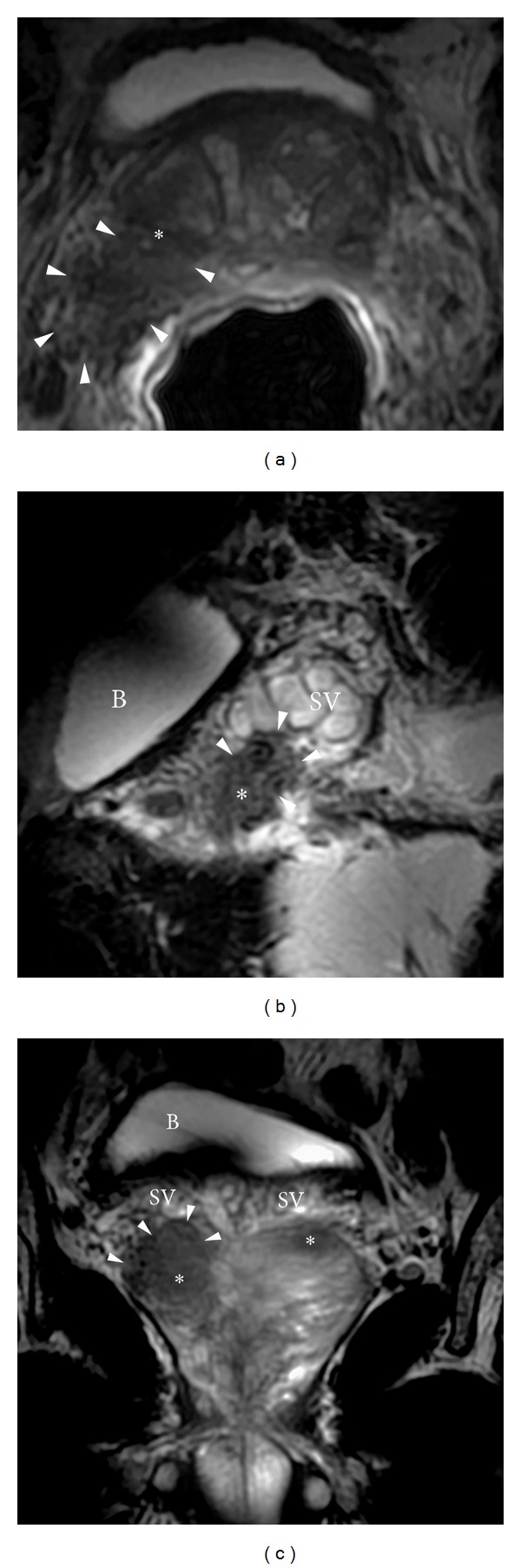
Prostate cancer with seminal vesicle invasion. (a) Axial T2WI showing a right posterolateral peripheral zone tumor (^∗^) which is contiguous with the adjacent seminal vesicle tubules (arrowheads). According to the TNM staging system, this would be consistent with at least a T3 tumor (Stage III). Prostate cancer with seminal vesicle invasion. (b) Sagittal T2WI showing a right posterolateral peripheral zone tumor (^∗^) which is contiguous with the adjacent seminal vesicle tubules (arrowheads). According to the TNM staging system, this would be consistent with at least a T3 tumor (Stage III). B: bladder; SV: normal-appearing seminal vesicle tubules. Prostate cancer with seminal vesicle invasion. (c) Coronal T2WI showing a right posterolateral peripheral zone tumor (^∗^) which is contiguous with the adjacent seminal vesicle tubules (arrowheads). An additional tumor is noted within the contralateral peripheral zone (also ^∗^). According to the TNM staging system, this would be consistent with at least a T3 tumor (Stage III). B: bladder; SV: normal-appearing seminal vesicle tubules. B: bladder.

Evaluation of the prostate capsule, seminal vesicles, and posterior bladder wall is also important when interpreting T2WI. Criteria for extracapsular extension are abutment: asymmetry, irregularity, and thickening of the neurovascular bundle; bulge, loss of capsule, and capsular enhancement; measurable extracapsular disease; and obliteration of the rectoprostatic angle (Figure 8) [2]. Abnormally low signal intensity expanding the vesicular lumen, focal thickening of the seminal vesicle wall, filling in of the prostate-seminal vesicle angle, and enhancement/restricted diffusion are suggestive of seminal vesicle invasion (Figure 9).

## 7. Prostate Cancer Staging

The classification systems used for the staging of prostate cancer are the TNM and Jewett systems. The Jewett system was initially introduced in 1975 and has since been modified [20]. In 1997, the American Joint Committee on Cancer (AJCC) and the Union for International Cancer Control (UICC) introduced a revised tumor, nodes, metastasis (TNM) system that employed the same broad T stage categories as the Jewett system but includes subcategories of T stage, such as a stage to describe patients diagnosed through PSA screening.

As per AJCC guidelines, regional nodes (N) are in the true pelvis, below the iliac artery bifurcations. They include hypogastric, obturator, iliac (internal, external), and sacral (lateral, presacral, and promontory). Distant lymph nodes are outside the confines of the true pelvis. Involvement of distant lymph nodes is classified as M1a.

The AJCC TNM system (7th edition) is shown in Tables 2 and 3.

One of the more important assessments is whether the tumor is confined to the gland (≤T2, organ-confined) or extends beyond the gland (≥T3, tumor extends beyond prostate). Generally, the ≥T3 tumors will show either extraglandular/extracapsular extension alone or combined with neurovascular bundle invasion and/or seminal vesicle invasion. The high spatial resolution and sharp demarcation of the prostate capsule in MR allow for assessments of such critical diagnostic staging criteria. Unfortunately, some cancers may show no evidence of extracapsular extension yet still represent an unconfined tumor. The detection of abnormal lymph nodes on MRI is currently limited to size assessment and enhancement. In general, lymph nodes over 5 mm in short axis dimension are regarded as suspicious. Bone metastases, which are sclerotic in prostate cancer, are identified as high signal foci on T2WI and low signal on fat-suppressed T1-weighted images. Such lesions should enhance upon administration of a gadolinium-based MR contrast agent.

## 8. Conclusion

MRI is the prime imaging modality in the evaluation of the prostate gland. The advent of improved field strength and introduction of multiparametric functional MR imaging has improved the detection of cancer and accuracy in the local staging of prostate cancer. Currently, MRI is the only modality that can be used to assess unilobar or bilobar disease, extracapsular extension and seminal vesicle invasion, and/or invasion of other adjacent structures such as the bladder, rectum, external sphincter, levator muscles, or the pelvic wall. Thus, understanding the normal MR anatomy of the prostate gland and the adjacent pelvic structures is paramount in subsequent imaging interpretation.

## Figures and Tables

**Table 1:** Table summarizing the histologic composition and embryologic origins of the various zones of the prostate gland.

	Central zone (CZ)	Transition zone (TZ)	Peripheral zone (PZ)
Volume of normal prostate (%)	25	5	70
Embryologic origin	Wolffian duct	Urogenital sinus	Urogenital sinus
Epithelium	Complex, large polygonal glands	Simple, small rounded glands	Simple, small rounded glands
Stroma	Compact	Compact	Loose
Origin of prostatic adenocarcinoma (%)	5	25	70
Benign prostatic hyperplasia (%)	—	100	—

**Table 2:** Prostate cancer TNM staging with histopathologic grade per AJCC 7th edition.

Prostate cancer	TNM staging
T (tumor)	TX: tumor cannot be assessed T0: no evidence of primary tumor
	T1: clinically nonapparent tumor not palpable nor visible by imaging T1a: tumor incidental histologic finding in 5% or less of tissue resected T1b: tumor incidental histologic finding in more than 5% of tissue resected T1c: tumor identified by needle biopsy (e.g., because of elevated PSA)
	T2: tumor confined within the prostate∗ T2a: tumor involves 50% or less of one lobe T2b: tumor involves more than 50% of one lobe but not both lobes T2c: tumor involves both lobes
	T3: tumor extends through the prostate capsule∗∗ T3a: extracapsular extension (unilateral or bilateral) T3b: tumor invades seminal vesicle(s)
	T4: tumor is fixed or invades adjacent structures other than seminal vesicles: bladder neck, external sphincter, rectum, levator muscles, and/or pelvic wall

N (node)	NX: regional lymph nodes were not assessed N0: no regional lymph node metastasis N1: metastasis in regional lymph node(s)

M (metastasis)	MX: distant metastasis (M)∗∗∗ cannot be assessed (not evaluated by any modality) M0: no distant metastasis M1: distant metastasis M1a: nonregional lymph node(s) M1b: bone(s) M1c: other site(s) with or without bone disease

Histopathologic grade	GX: grade (G) cannot be assessed G1: well-differentiated (slight anaplasia, Gleason score of 2–4) G2: moderately differentiated (moderate anaplasia, Gleason score of 5-6) G3-4: poorly differentiated or undifferentiated (marked anaplasia, Gleason score of 7–10)

∗Tumor that is found in one or both lobes by needle biopsy but is not palpable or reliably visible by imaging is classified as T1c.

∗∗Invasion into the prostatic apex or into (but not beyond) the prostatic capsule is classified as T2 not T3.

∗∗∗When more than one metastasis site is present, the most advanced category pM1c is used.

**Table 3:** AJCC 7th edition stage groupings.

Stage	TNM categories
Stage 1	T1a, N0, M0, and G1

Stage II	T1a, N0, M0, and G2–4
	T1b, N0, M0, and any G
	T1c, N0, M0, and any G
	T1, N0, M0, and any G
	T2, N0, M0, and any G

Stage III	T3, N0, M0, and any G

Stage IV	T4, N0, M0, and any G
	Any T, N1, M0, and any G
	Any T, any N, M1, and any G
